# West Nile Virus–Associated Hemophagocytic Lymphohistiocytosis, Switzerland

**DOI:** 10.3201/eid3112.250776

**Published:** 2025-12

**Authors:** Clemente Lascano, Lena Groenendijk, Benjamin Bruno, Enrico Meduri, Ariane Malclès, Florian Laubscher, Francisco Javier Pérez-Rodriguez, Manuel Schibler, Christophe Marti, Aude Nguyen

**Affiliations:** Geneva University Hospitals, Geneva, Switzerland (C. Lascano, L. Groenendijk, E. Meduri, A. Malclès, F. Laubscher, F.J. Pérez-Rodriguez, M. Schibler, C. Marti, A. Nguyen); Centre Hospitalier de Wallonie Picard, Tournai, Belgium (B. Bruno); University of Geneva, Geneva (A. Malclès, M. Schibler)

**Keywords:** West Nile virus, viruses, hemophagocytic lymphohistiocytosis, high-throughput sequencing, vector-borne infections, meningitis/encephalitis, mosquito-borne, Switzerland

## Abstract

A 62-year-old patient was hospitalized in Geneva, Switzerland, with an atypical manifestation of West Nile virus infection. Initially, he sought care for febrile diarrhea and vomiting; his condition deteriorated and hemophagocytic lymphohistiocytosis and meningoencephalitis developed. Corticosteroids improved his condition. We used high-throughput sequencing and ophthalmologic findings to diagnose West Nile virus.

West Nile virus (WNV) is an enveloped, single-stranded positive-sense RNA virus belonging to the genus *Orthoflavivirus*, family *Flaviviridae*. First isolated from a patient in Uganda in 1937, this mosquitoborne virus has spread worldwide, causing sporadic infections and outbreaks on every continent except Antarctica (*1*). Birds, the natural reservoir, act as amplifying hosts and play a major role in the spread of WNV (*2*). Since the late 1990s, an increasing number of outbreaks have been reported in Southern and Central Europe, suggesting a trend to an endemic presence in various regions of Europe (*3*). That trend might be partly explained by modifications of ecosystems and climate change. Rising temperature and fluctuating rainfall can influence mosquito activity, bird migration patterns and their abundance and of consequence, the expansion of vector habitat that can contribute to increased virus transmission (*4*,*5*). To date, 9 phylogenetic lineages of WNV have been identified, of which only lineages 1 and 2 are associated with major outbreaks in humans (*3*,*4*).

The WNV incubation period ranges from 2–15 days after a mosquito bite. Approximately 20% of infected persons will experience an acute systemic illness, known as West Nile fever, characterized generally by fever, malaise, headaches, myalgias, arthralgias, maculopapular rash, and gastrointestinal symptoms. Neuroinvasive disease will develop in <1% of infected persons, manifesting as meningitis, encephalitis, or acute flaccid paralysis, a poliomyelitis-like syndrome (*6*). Among those with neuroinvasive disease, the mortality rate ranges from 10–30%, which represents <0.1% of all infected cases (*1*,*6*).

Hemophagocytic syndromes or hemophagocytic lymphohistiocytosis (HLH) are rare, underdiagnosed, and often life-threatening conditions because of an overstimulation of the immune system, leading to systemic inflammation (activated cytotoxic T cells and natural killer cells), cytokine storm, and multiorgan failure. Those syndromes can be divided into primary HLH, associated with genetic factors and usually seen in children, and secondary HLH, also called reactive hemophagocytic syndrome (RHS), which can be triggered by malignancy, autoimmune diseases, or infections (*7*,*8*). The most common infectious triggers are herpes viruses, in the case of primary infection or reactivation, although many other viruses, such as HIV, hepatitis viruses, parvovirus B19, and influenza virus, have been reported (*9*). In adults, the average age of diagnosis is 50, and more women are affected than men (*8*). Diagnosis is currently made on the basis of various diagnostic criteria, such as HLH-2004 criteria (commonly used) and the HScore (specifically developed for adult RHS) (*10*). Those criteria include a constellation of clinical manifestations and biologic or cytological findings: fever, hepatosplenomegaly, cytopenia, elevated plasmatic ferritin, high lactate dehydrogenase and triglycerides, low fibrinogen, hepatic cytolysis and cholestasis, and hemophagocytosis in bone marrow biopsy (*7*,*8*). However, none of those criteria are pathognomonic alone. An HLH diagnosis can be made even in the absence of hemophagocytosis in bone marrow biopsy (*6*). We report a case of WNV-associated hemophagocytic lymphohistiocytosis in a man in Switzerland.

## The Case

A 62-year-old man with a history of prostate adenocarcinoma under close follow-up by urologists since 2022 was admitted to Geneva University Hospitals (Geneva, Switzerland) in August 2024 with complaints of a 48-hour history of fever, watery diarrhea, and vomiting. He had recently returned from a trip to Toulon on the south coast of France. He also reported moderate bitemporal headache for 1 week without photophobia or phonophobia.

In the emergency department, the patient was febrile with signs of septic shock but had an otherwise unremarkable physical examination. Initial workup revealed thrombocytopenia (58 × 10^9^ cells/L, reference range 150–400 × 10^9^ cells/L), lymphocytopenia (0.12 × 10^9^ cells/L, reference 4–11 × 10^9^ cells/L), elevated C-reactive protein (391 mg/L, reference <0.9 mg/L), and acute kidney injury (KDIGO stage 2 [https://kdigo.org/guidelines/acute-kidney-injury]). Abdominal contrast-enhanced computed tomography scan revealed hepatosplenomegaly. We admitted the patient to a monitored care unit for presumed bacterial sepsis of enteric origin and provide empirical antimicrobial therapy (ceftriaxone 2 g/d and metronidazole 500 mg/8 h), along with intensive intravenous fluid therapy. We performed a microbiological diagnostic workup, including serology for viral hepatitis, cytomegalovirus, Epstein-Barr virus, HIV, parvovirus B19, syphilis, and leptospirosis; all results were negative for acute infection. Results of a gastrointestinal multiplex molecular assay were positive for enteropathogenic *Escherichia coli*, which we believed reflected colonization.

**Figure 1:**
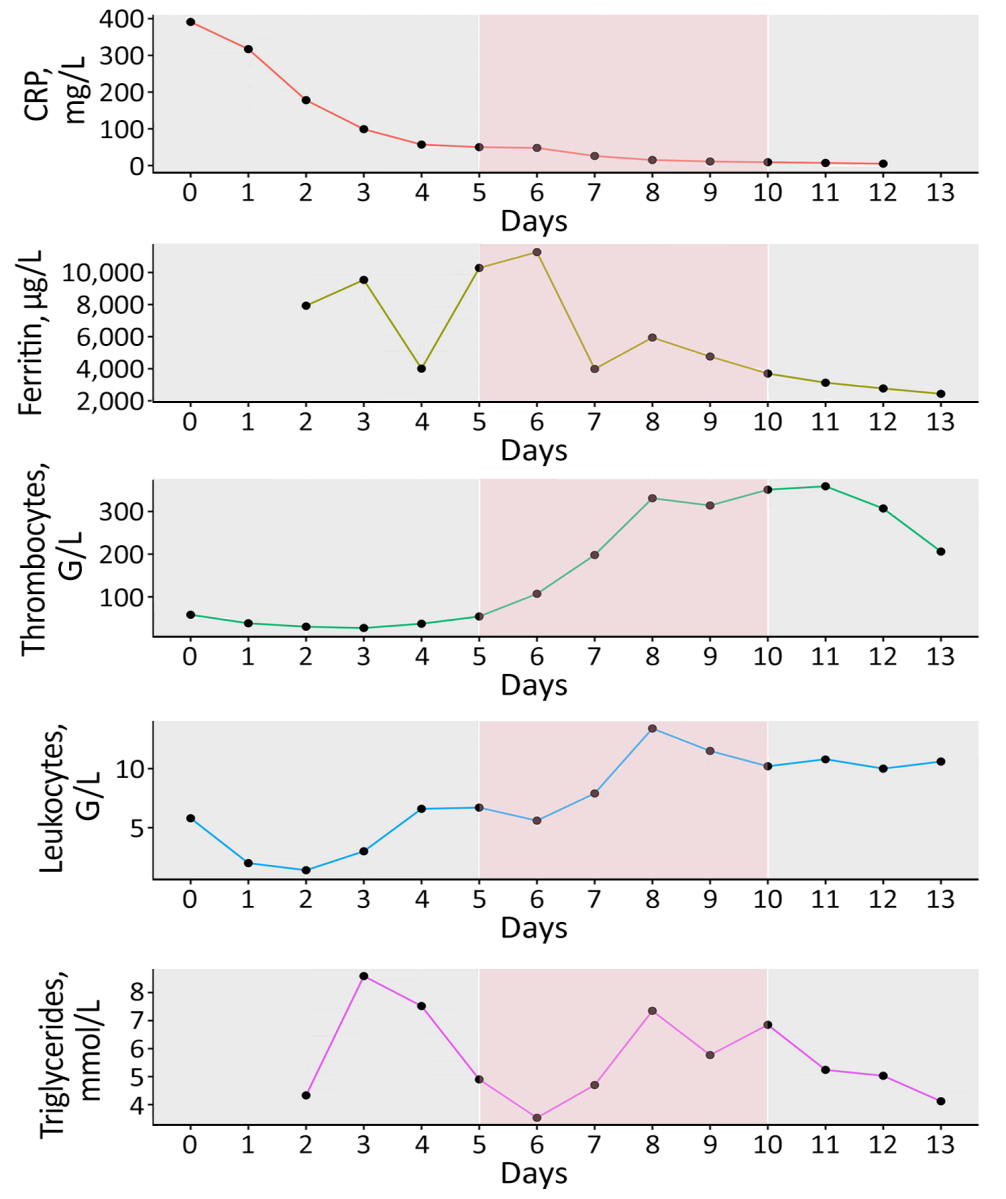
Evolution of laboratory data for a patient with West Nile virus–associated hemophagocytic lymphohistiocytosis during hospitalization, Geneva, Switzerland. Results include C-reactive protein, ferritin, thrombocytes, leukocytes, and triglycerides. Pink shading indicates corticosteroid treatment.

**Figure 2:**
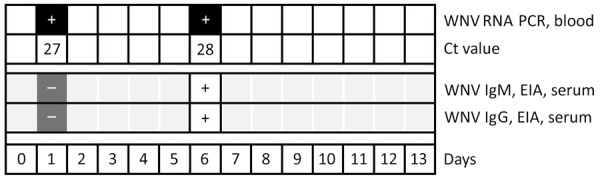
WNV testing results of a patient with WNV-associated hemophagocytic lymphohistiocytosis during hospitalization, Geneva, Switzerland. Ct, cycle threshold; EIA, enzyme immunoassay; WNV, West Nile virus.

During the patient’s hospitalization, a persistent fever developed and kidney failure worsened; profound pancytopenia, hyperferritinemia (11,272 mcg/L, reference 12–200 mcg/L), hypertriglyceridemia (7.35 mmol/L, reference <2 mmol/L), and isolated aspartate aminotransferase elevation occurred (Figures 1, 2). Hematology consultation led to the suspicion of an HLH secondary to a concurrent yet undetermined infection. That suspicion was supported by an HScore of 244 points (high temperature; organomegaly; cytopenia; elevated ferritin, triglycerides, and aspartate aminotransferase; reference fibrinogen levels; and absence of hemophagocytosis features on bone marrow), translating to an HLH diagnostic probability >99%. Total body positron emission tomography–computed tomography revealed no malignancy. Bone marrow biopsy showed no evidence of hematological neoplasia or hemophagocytosis. The infectious workup showed no herpetic infection or reactivation; blood PCR results were negative for herpes simplex virus 1 and 2, human herpesvirus 6, Epstein-Barr virus, and cytomegalovirus. Dengue virus was also not detected.

On day 4 of hospitalization, the patient experienced altered mental status, psychomotor slowing, and disorientation. Cerebrospinal fluid (CSF) analysis revealed mixed pleocytosis (leukocyte count 327 mol/L, 48% lymphocytes and 46% polymorphonuclear cells), proteinorachia, and hypoglycorrhachia. Suspecting neurolisteriosis, we changed the antimicrobial therapy to meropenem (2 g/12 h). Results of a multiplex molecular panel for meningitis and encephalitis and a CSF culture were negative. Brain MRI showed mesial temporal hyperintensity, and electroencephalogram indicated no epileptic activity. Soluble CD25 serum concentration on day 5 of hospitalization was elevated at 4 ng/mL (reference <2 ng/mL). We started the patient on dexamethasone (20 mg 1×/d) for possible HLH-related central nervous system involvement; the treatment led to fever resolution, slow neurologic status improvement, and normalization of ferritin and triglyceride values (Figures 1, 2). We discontinued meropenem and continued corticotherapy for a total of 5 days. Ophthalmological evaluation for new onset myodesopsia revealed uveitis and scattered chorioretinal lesions with a targetoid appearance, findings highly suggestive of WNV chorioretinitis (Appendix Figure 1).

An unbiased high-throughput sequencing performed on the initial serum sample to identify a viral HLH trigger identified WNV sequences (4 reads on 2 separate genomic regions, enabling classification in WNV lineage 2) (Appendix Figure 2). We isolated RNA by using TRIzol (Invitrogen, https://www.invitrogen.com) according to the manufacturer’s instructions. We prepared libraries by using the TruSeq (Illumina, https://www.illumina.com) total RNA preparation protocol and then sequenced on a MiSeq platform (Illumina) by using the 2 × 75 bp paired-end protocol. We analyzed reads by using a bioinformatics pipeline designed to detect all vertebrate viruses on the basis of the Virosaurus database as previously described (*11*). Subsequent serology and blood PCR confirmed the viral diagnosis, whereas CSF WNV reverse transcription PCR was negative.

## Conclusions

We report an atypical manifestation of WNV infection with features of meningoencephalitis, ophthalmological involvement, and secondary HLH. Of note, the pathognomic chorioretinal findings provided the first hint pointing toward a viral etiology. The infection was likely acquired on the south coast of France, where multiple infections were reported at the time. Although cases of HLH secondary to other flaviviruses such as dengue virus have been reported, RHS triggered by WNV infection is unusual (*12*,*13*). The diagnosis of RHS was established despite the absence of hemophagocytosis in the bone marrow biopsy, consistent with current diagnostic standards (*8*,*10*). Corticosteroid treatment led to clinical improvement. Because of the ongoing effects of climate change and the increasing incidence of WNV infections, clinicians should be aware of potential clinical manifestations and WNV’s possible complications, such as secondary HLH, which might affect patient prognosis.

Appendix Additional information about West Nile virus–associated hemophagocytic lymphohistiocytosis, Switzerland.
