# Supplementary material for: West Nile Virus–Associated Hemophagocytic Lymphohistiocytosis, Switzerland
Source: Emerg Infect Dis. 2025 Dec;31(12):2289–92. doi: 10.3201/eid3112.250776 (PMC12782237; doi:10.3201/eid3112.250776)
Supplement: Appendix — Additional information about West Nile virus–associated hemophagocytic lymphohistiocytosis, Switzerland. [file 25-0776-Techapp-s1.pdf]

Article DOI: https://doi.org/10.3201/eid3112.250776

EID cannot ensure accessibility for supplementary materials supplied by authors. Readers who have difficulty accessing supplementary content should contact the authors for assistance.

West Nile Virus–Associated Hemophagocytic Lymphohistiocytosis, Switzerland

Appendix

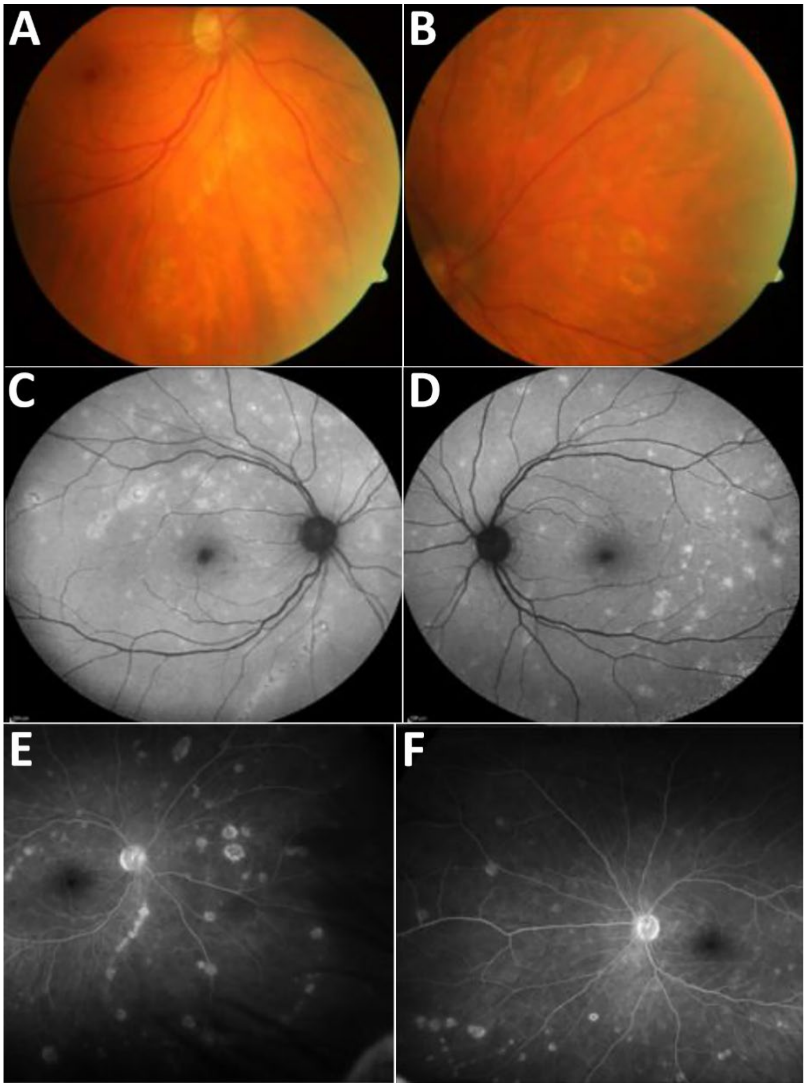

Appendix Figure 1. Fundoscopy, fundus autofluorescence, and fluorescein angiography. Multifocal chorioretinal lesions with target-like appearance.

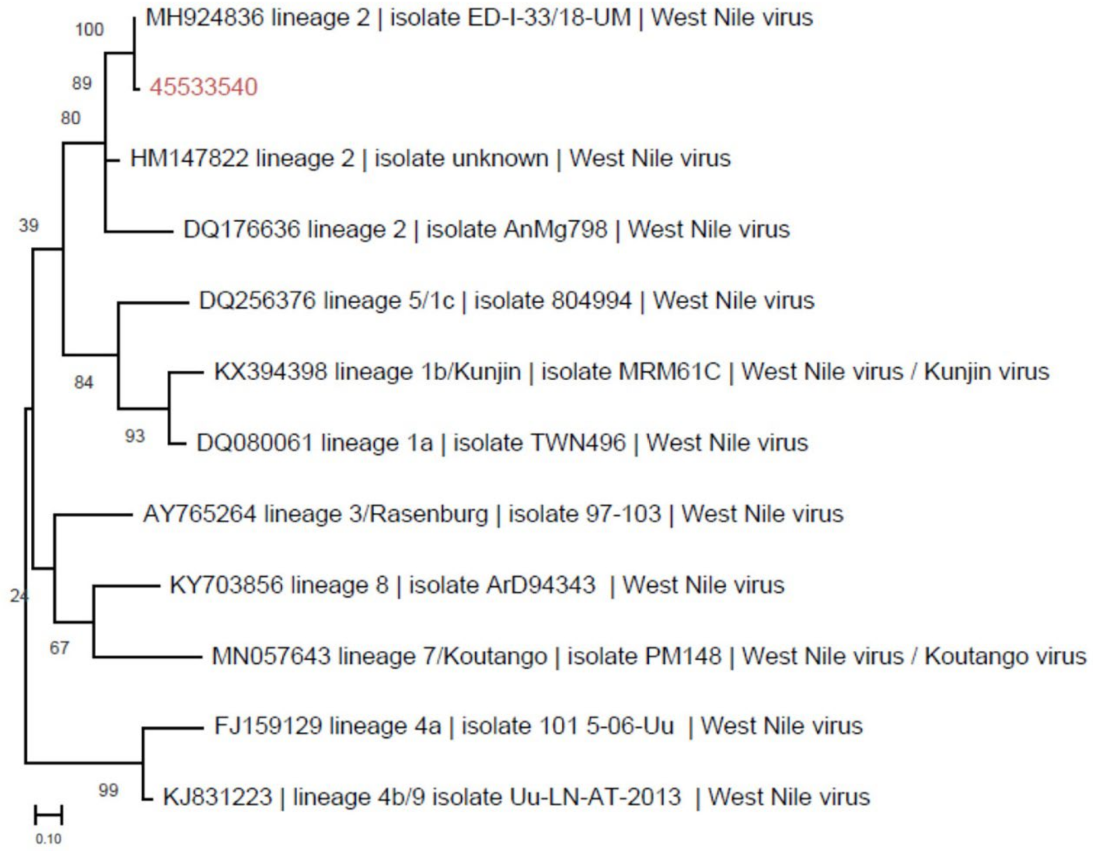

Appendix Figure 2. Phylogenetic tree. Unrooted phylogenetic tree of West Nile virus lineages inferred using the Maximum Likelihood method under the General Time Reversible model. The scale bar indicates the number of substitutions per site. 45533540 corresponds to the patient’s isolate.
